# Author Correction: Microbiome disturbance and resilience dynamics of the upper respiratory tract during influenza A virus infection

**DOI:** 10.1038/s41467-020-17020-y

**Published:** 2020-06-16

**Authors:** Drishti Kaul, Raveen Rathnasinghe, Marcela Ferres, Gene S. Tan, Aldo Barrera, Brett E. Pickett, Barbara A. Methe, Suman R. Das, Isolda Budnik, Rebecca A. Halpin, David Wentworth, Mirco Schmolke, Ignacio Mena, Randy A. Albrecht, Indresh Singh, Karen E. Nelson, Adolfo García-Sastre, Chris L. Dupont, Rafael A. Medina

**Affiliations:** 1 J. Craig Venter Institute, 4120 Capricorn Lane, La Jolla, CA 92037 USA; 2 Departmento de Enfermedades Infecciosas e Inmunología Pediátrica, Facultad de Medicina, Pontificia Universidad Católica de Chile, Santiago, Chile; 3 Department of Infectious Diseases, University of California San Diego, La Jolla, CA 92037 USA; 4 Millennium Institute on Immunology and Immunotherapy, Santiago, Chile; 5 J. Craig Venter Institute, 9704 Medical Center Drive, Rockville, MD 20850 USA; 6 Microbiology & Molecular Biology, Brigham Young University, Provo, UT USA; 7 Department of Medicine, University of Pittsburgh, Pittsburgh, PA 15213 USA; 8 Department of Microbiology, Global Health and Emerging Pathogens Institute, Icahn School of Medicine at Mount Sinai, New York, NY 10029 USA; 9 Department of Medicine, Icahn School of Medicine at Mount Sinai, New York, NY 10029 USA; 10 Present Address: National Center for Immunization and Respiratory Diseases, Centers for Disease Control and Prevention, Atlanta, GA USA; 11 Present Address: Department of Microbiology and Molecular Medicine, University of Geneva, Geneva, Switzerland

**Keywords:** Microbiome, Influenza virus, Influenza virus, Translational research

Correction to: *Nature Communications* 10.1038/s41467-020-16429-9, published online 21 May 2020.

The original version of this Article contained an error in the spelling of the author Suman R. Das, which was incorrectly given as Suman Das. This has now been corrected in both the PDF and HTML versions of the Article.

