# Supplementary material for: Microbiome disturbance and resilience dynamics of the upper respiratory tract during influenza A virus infection
Source: Nat Commun. 2020 May 21;11:2537. doi: 10.1038/s41467-020-16429-9 (PMC7242466; doi:10.1038/s41467-020-16429-9)
Supplement: Supplementary file 1 — Supplementary Information [file 41467_2020_16429_MOESM1_ESM.pdf]

Supplemental information for

Microbiome disturbance and resilience dynamics of the upper respiratory tract during influenza A virus infection

Drishti Kaul[1,8], Raveen Rathnasinghe[2,8], Marcela Ferres[2], Gene S. Tan[1,3], Aldo Barrera[2,4], Brett E. Pickett[5], Barbara A. Methe[5], Suman Das[5], Isolda Budnik[2], Rebecca Halpin[5], David Wentworth[5b], Mirco Schmolke[6c], Ignacio Mena[6], Randy A. Albrecht[6], Indresh Singh[5], Karen E. Nelson[5], Adolfo Garcia-Sastre[6,7], Chris L. Dupont[1*], Rafael A. Medina[2,4,6*].

[1]J. Craig Venter Institute, 4120 Capricorn Lane, La Jolla, CA 92037, USA.

[2]Departmento de Enfermedades Infecciosas e Inmunología Pediátrica, Facultad de Medicina, Pontificia Universidad Católica de Chile, Santiago, Chile.

[3] Department of Infectious Diseases, University of California San Diego, La Jolla, CA 92037, USA

[4]Millennium Institute on Immunology and Immunotherapy, Santiago, Chile

[5]J. Craig Venter Institute, 9704 Medical Center Drive, Rockville, Maryland 20850, 14 USA.

[6]Department of Microbiology, Global Health and Emerging Pathogens Institute, Icahn School of Medicine at Mount Sinai, New York, NY 10029, USA.

[7]Department of Medicine, Icahn School of Medicine at Mount Sinai, New York, NY 10029, USA.

[8]These authors contributed equally to this work

[b] Present address: National Center for Immunization and Respiratory Diseases, Centers for Disease Control and Prevention, Atlanta, GA, USA.

[c] Present address: Department of Microbiology and Molecular Medicine, University of Geneva, Switzerland

*Corresponding author: E-mail: rmedinai@uc.cl, cdupont@jcvi.org

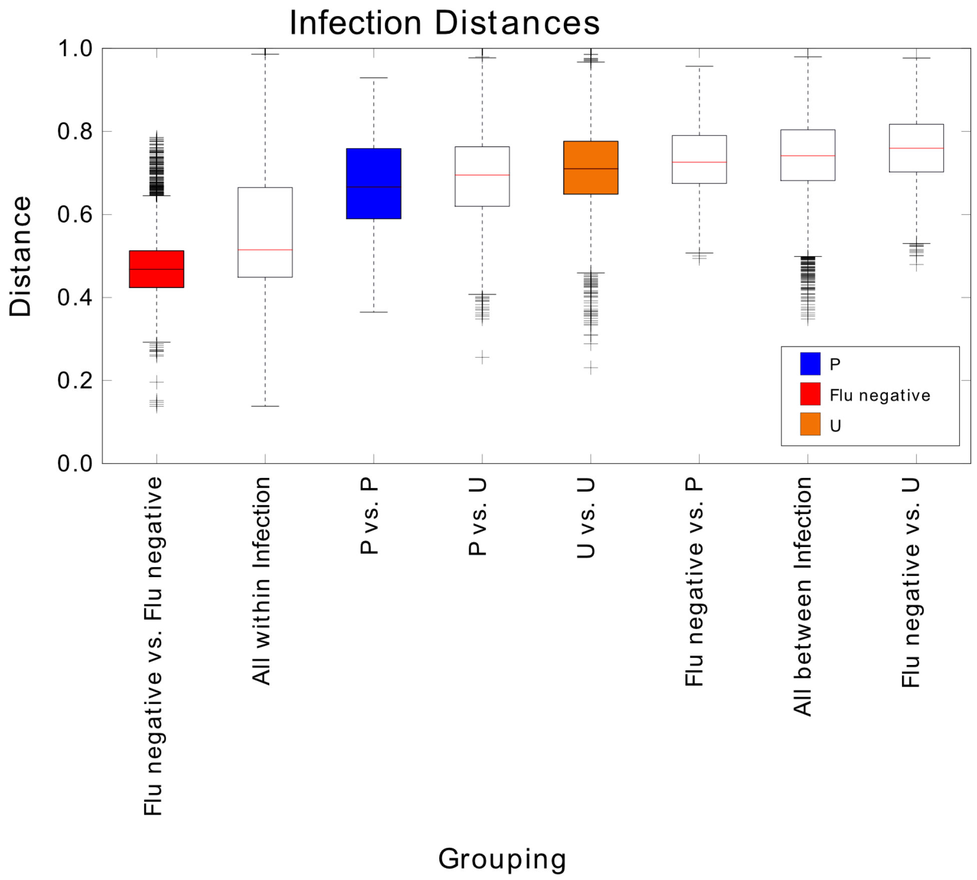

Supplementary Figure 1. Diversity distance analyses of the microbiome of infected and uninfected humans. Box and whisker plots for beta diversity distances within and between influenza types for the human samples (P: Influenza positive, U: Influenza unknown, Flu negative). The boxplots represent the diversity between the different infection types. All the distances were calculated using the Bray-Curtis metric. The box represents the interquartile range, the red line in the box indicates median for each of the sample groupings and the error bars represent standard deviation. Dotted whiskers outside the box extend from the highest to the lowest observation represented in the plot. Source data are provided as a Source Data file.

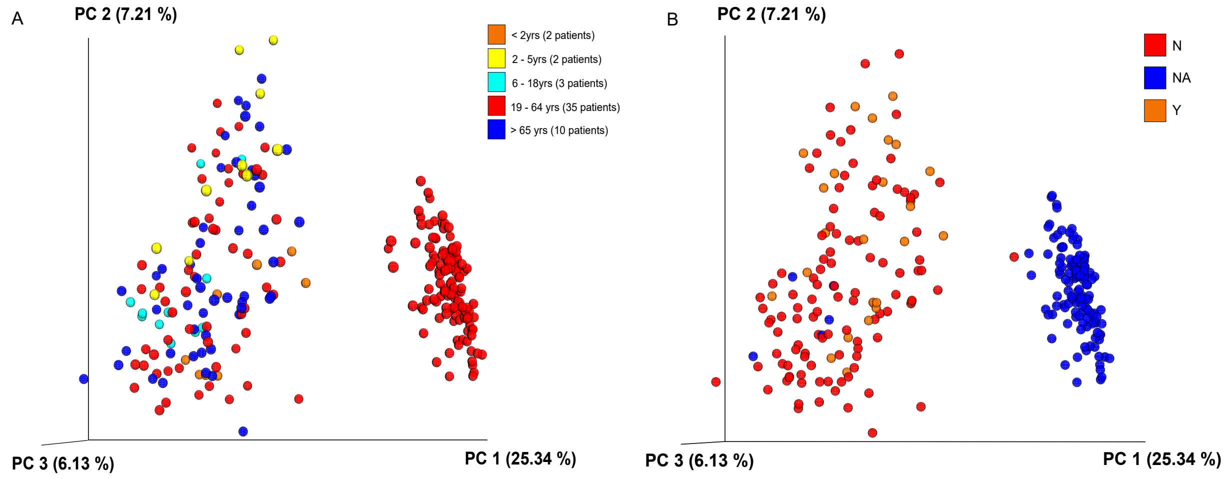

Supplementary Figure 2: Microbiome stratification for human patients according to two clinical factors. Clustering of data points for all human patients shown according to A) different age groupings and B) Vaccination status (NA: Not available/ applicable; Y: Yes; N: No). Source data are provided as a Source Data file.

(a) (b) (c) (d)

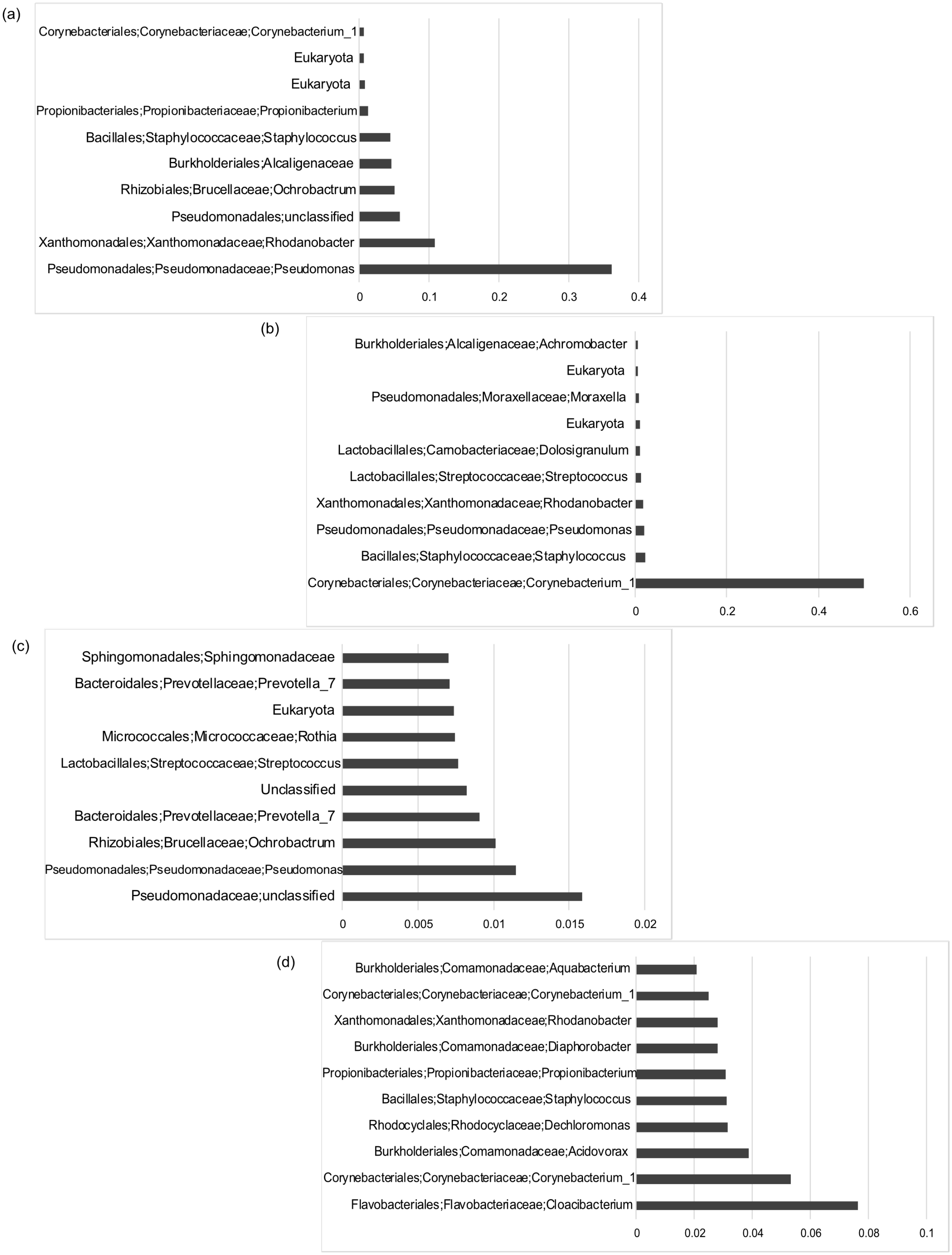
(a)
Corynebacteriales;Corynebacteriaceae;Corynebacterium_1
Eukaryota
Eukaryota
Propionibacteriales;Propionibacteriaceae;Propionibacterium
Bacillales;Staphylococcaceae;Staphylococcus
Burkholderiales;Alcaligenaceae
Rhizobiales;Brucellaceae;Ochrobactrum
Pseudomonadales;unclassified
Xanthomonadales;Xanthomonadaceae;Rhodanobacter
Pseudomonadales;Pseudomonadaceae;Pseudomonas
0 0.1 0.2 0.3 0.4
(b)
Burkholderiales;Alcaligenaceae;Achromobacter
Eukaryota
Pseudomonadales;Moraxellaceae;Moraxella
Eukaryota
Lactobacillales;Carnobacteriaceae;Dolosigranulum
Lactobacillales;Streptococcaceae;Streptococcus
Xanthomonadales;Xanthomonadaceae;Rhodanobacter
Pseudomonadales;Pseudomonadaceae;Pseudomonas
Bacillales;Staphylococcaceae;Staphylococcus
Corynebacteriales;Corynebacteriaceae;Corynebacterium_1
0 0.2 0.4 0.6
(c)
Sphingomonadales;Sphingomonadaceae
Bacteroidales;Prevotellaceae;Prevotella_7
Eukaryota
Micrococcales;Micrococcaceae;Rothia
Lactobacillales;Streptococcaceae;Streptococcus
Unclassified
Bacteroidales;Prevotellaceae;Prevotella_7
Rhizobiales;Brucellaceae;Ochrobactrum
Pseudomonadales;Pseudomonadaceae;Pseudomonas
Pseudomonadaceae;unclassified
0 0.005 0.01 0.015 0.02
(d)
Burkholderiales;Comamonadaceae;Aquabacterium
Corynebacteriales;Corynebacteriaceae;Corynebacterium_1
Xanthomonadales;Xanthomonadaceae;Rhodanobacter
Burkholderiales;Comamonadaceae;Diaphorobacter
Propionibacteriales;Propionibacteriaceae;Propionibacterium
Bacillales;Staphylococcaceae;Staphylococcus
Rhodocyclales;Rhodocyclaceae;Dechloromonas
Burkholderiales;Comamonadaceae;Acidovorax
Corynebacteriales;Corynebacteriaceae;Corynebacterium_1
Flavobacteriales;Flavobacteriaceae;Cloacibacterium
0 0.02 0.04 0.06 0.08 0.1

42

Supplementary Figure 3. Relative abundance for the top ten bacterial families in the URT among infected and uninfected human subjects. The relative abundance values for the most prevalent bacterial families among the three infected ecostates (a, b, and c,) and the one uninfected (d) ecostate from human samples based on the Bayesian posterior predictive probabilities from the Infinite Dirichlet Multinomial mixture Models run over 2000 iterations (top to bottom, (a)-(d)). Source data are provided as a Source Data file.

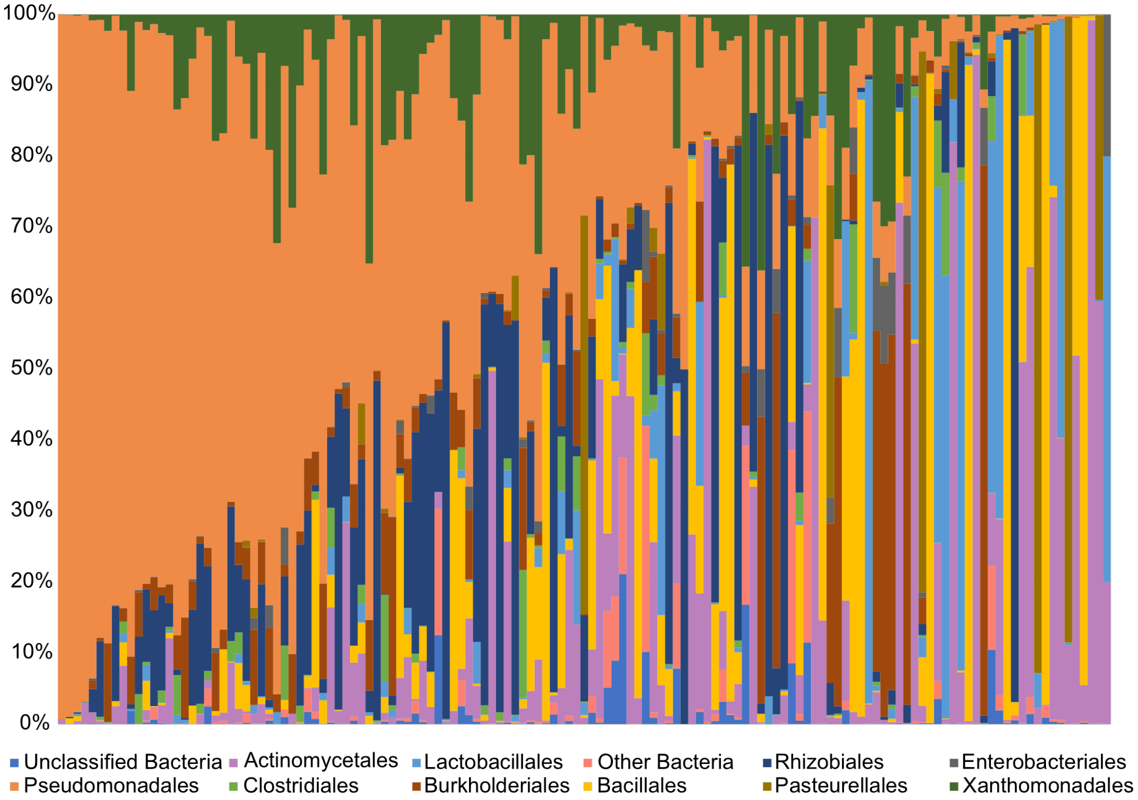

Supplementary Figure 4. Comprehensive taxonomic breakdown for influenza-infected human subjects. The plot summarizes the relative abundances at the order level for taxonomic groups that are present in greater than 1% of the samples. Each vertical column is an individual subject. Source data are provided as a Source Data file.

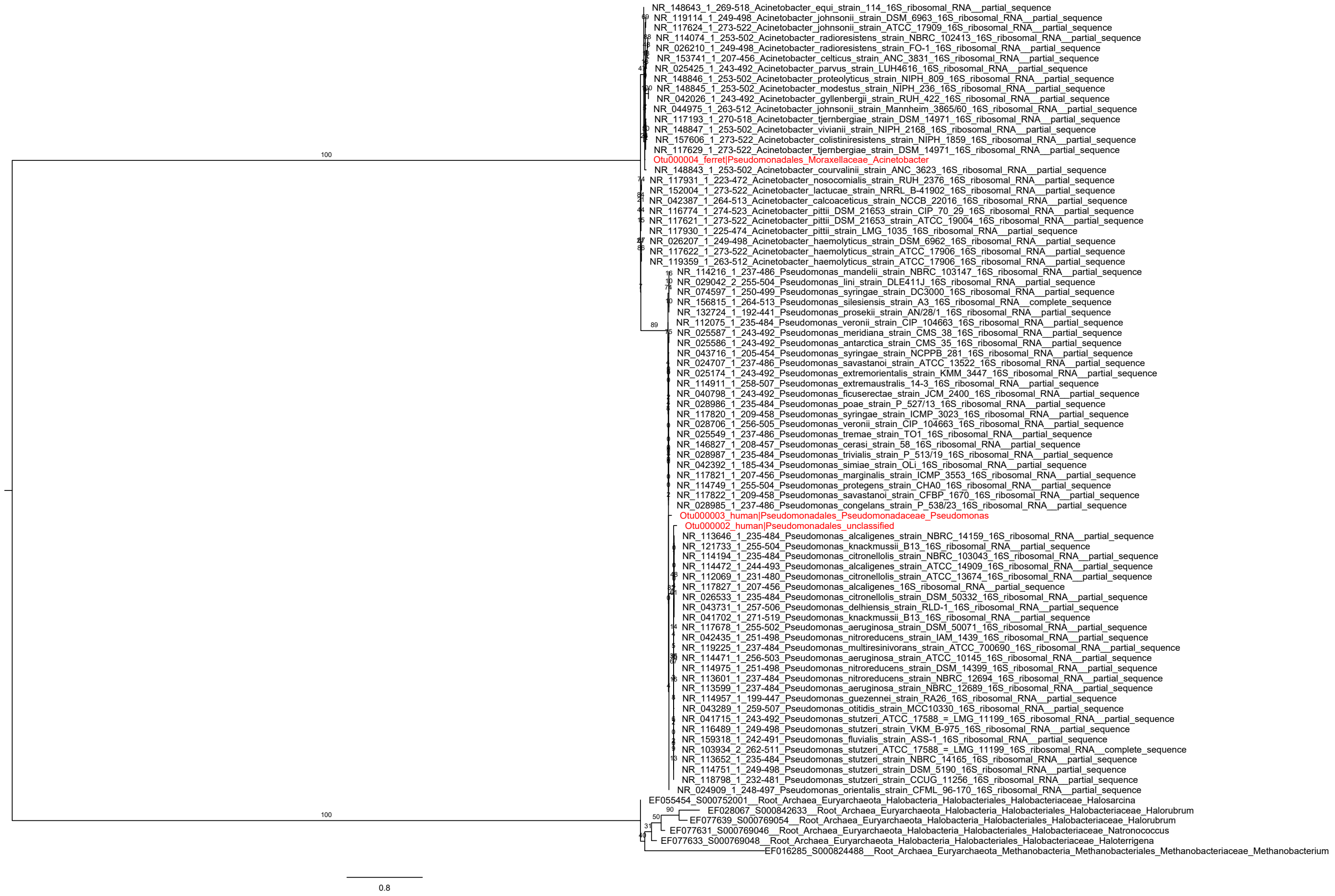

Supplementary Figure 5: Phylogenetic inference of the diagnostic *Pseudomonadales* OTUs relative to reference sequences from cultivated strains. Source data are provided as a Source Data file.

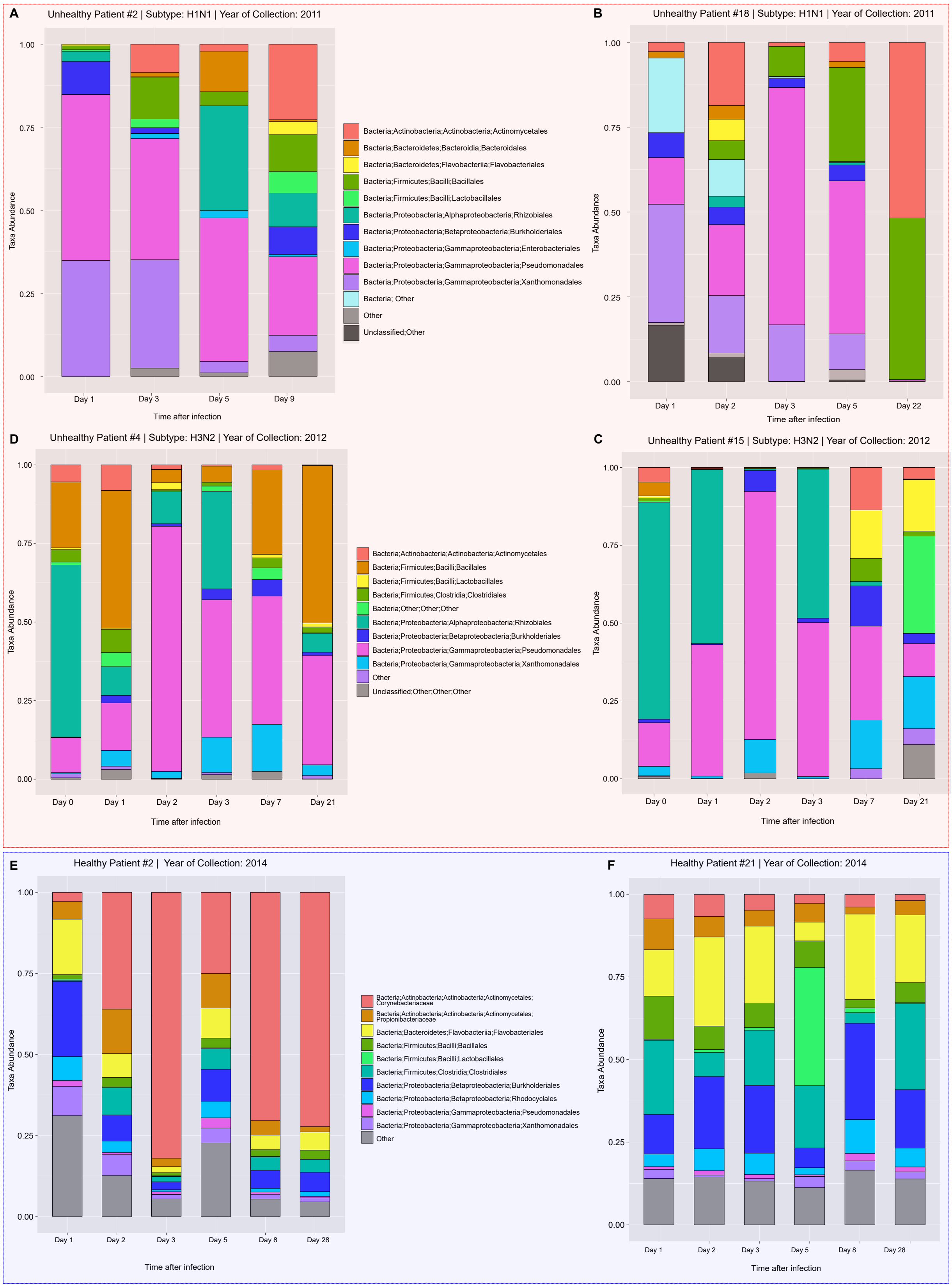
A
Unhealthy Patient #2 | Subtype: H1N1 | Year of Collection: 2011
B
Unhealthy Patient #18 | Subtype: H1N1 | Year of Collection: 2011
Taxa Abundance
Time after infection
Day 1
Day 3
Day 5
Day 9
Day 2
Day 22
Bacteria;Actinobacteria;Actinobacteria;Actinomycetales
Bacteria;Bacteroidetes;Bacteroidia;Bacteroidales
Bacteria;Bacteroidetes;Flavobacteriia;Flavobacteriales
Bacteria;Firmicutes;Bacilli;Bacillales
Bacteria;Firmicutes;Bacilli;Lactobacillales
Bacteria;Proteobacteria;Alphaproteobacteria;Rhizobiales
Bacteria;Proteobacteria;Betaproteobacteria;Burkholderiales
Bacteria;Proteobacteria;Gammaproteobacteria;Enterobacteriales
Bacteria;Proteobacteria;Gammaproteobacteria;Pseudomonadales
Bacteria;Proteobacteria;Gammaproteobacteria;Xanthomonadales
Bacteria; Other
Other
Unclassified;Other
D
Unhealthy Patient #4 | Subtype: H3N2 | Year of Collection: 2012
C
Unhealthy Patient #15 | Subtype: H3N2 | Year of Collection: 2012
Day 0
Day 7
Day 21
Bacteria;Actinobacteria;Actinobacteria;Actinomycetales
Bacteria;Firmicutes;Bacilli;Bacillales
Bacteria;Firmicutes;Bacilli;Lactobacillales
Bacteria;Firmicutes;Clostridia;Clostridiales
Bacteria;Other;Other;Other
Bacteria;Proteobacteria;Alphaproteobacteria;Rhizobiales
Bacteria;Proteobacteria;Betaproteobacteria;Burkholderiales
Bacteria;Proteobacteria;Gammaproteobacteria;Pseudomonadales
Bacteria;Proteobacteria;Gammaproteobacteria;Xanthomonadales
Other
Unclassified;Other;Other;Other
E
Healthy Patient #2 | Year of Collection: 2014
F
Healthy Patient #21 | Year of Collection: 2014
Day 8
Day 28
Bacteria;Actinobacteria;Actinobacteria;Actinomycetales; Corynebacteriaceae
Bacteria;Actinobacteria;Actinobacteria;Actinomycetales; Propionibacteriaceae
Bacteria;Bacteroidetes;Flavobacteriia;Flavobacteriales
Bacteria;Firmicutes;Bacilli;Bacillales
Bacteria;Firmicutes;Bacilli;Lactobacillales
Bacteria;Firmicutes;Clostridia;Clostridiales
Bacteria;Proteobacteria;Betaproteobacteria;Burkholderiales
Bacteria;Proteobacteria;Betaproteobacteria;Rhodocyclales
Bacteria;Proteobacteria;Gammaproteobacteria;Pseudomonadales
Bacteria;Proteobacteria;Gammaproteobacteria;Xanthomonadales
Other
0.00
0.25
0.50
0.75
1.00

Supplementary Figure 6. Comprehensive temporal taxonomic breakdown for human subjects. The plot summarizes the relative taxonomic abundances at the order level across all timepoints for taxonomic groups that are present in greater than 1% of the four influenza infected subjects (2 for each virus subtype, A-D clockwise) and 2 healthy subjects (E-F). Pseudomonadales (pink) is prevalent among the infected individuals (to 4), whereas inconsistent taxa are seen among the healthy control individuals (bottom 2). Source data are provided as a Source Data file.

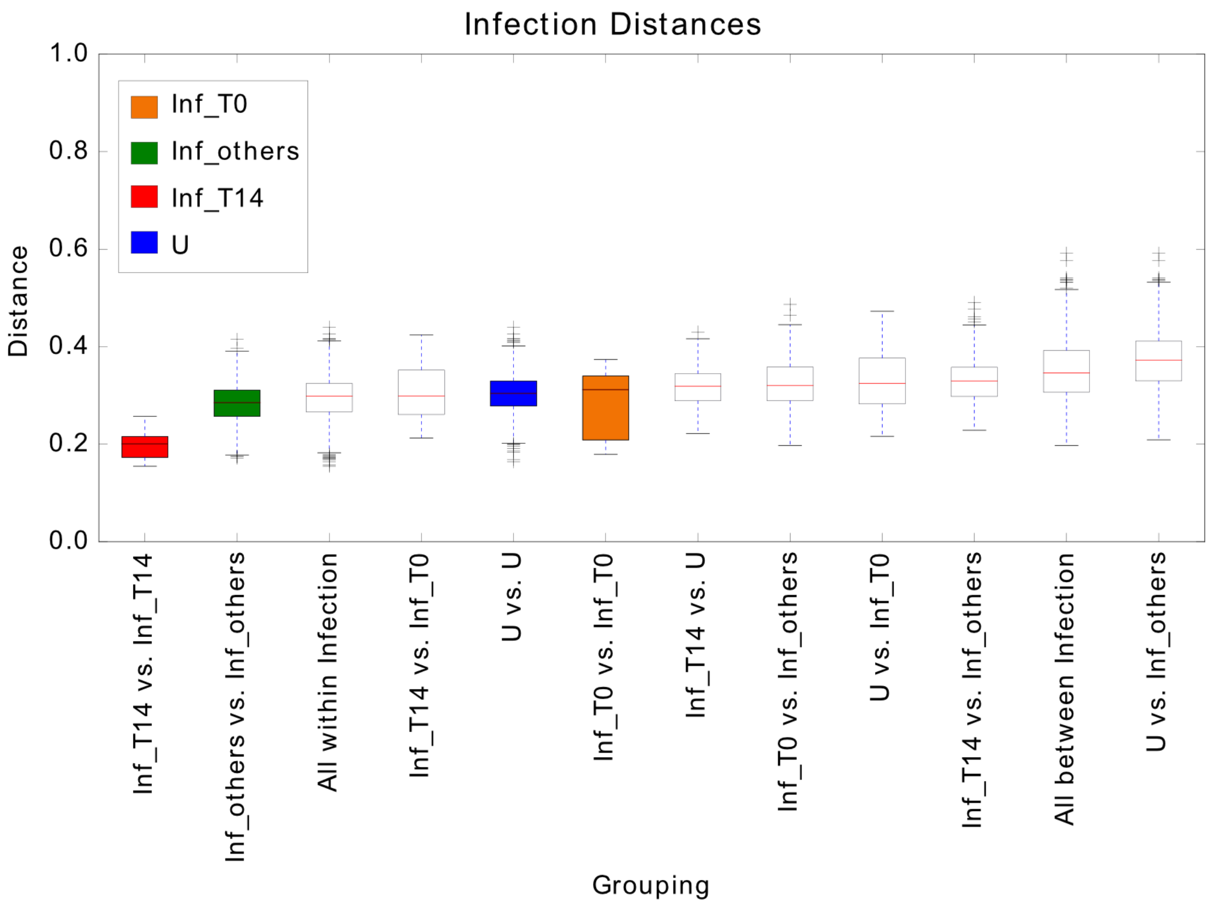

Supplementary Figure 7. Diversity distance analyses of the microbiome of infected and uninfected ferrets. Box and whisker plots for beta diversity distances within and between influenza types for samples obtained for groups of 7 Infected (grouped by time points as Inf_T0, Inf_others and Inf_T14) and 7 Uninfected (U) ferrets. The boxplots, sorted by median, represent the diversity between and within the different infection types; boxplots identifying individual time point-specific within diversity have been colored. Diversity within infected ferrets from T=14 (red) being the least, and diversity between all uninfected (U) and infected ferrets from the acute viral timepoints (T=1,3,5,7 dpi) being the highest, followed closely by diversity between all infection states (all Infected vs all Uninfected) being the second highest. All the distances were calculated using the Bray-Curtis metric. The box represents the interquartile range, the

horizontal line in the box indicates median for each of the sample groupings and the error bars represent standard deviation. Dotted whiskers outside the box extend from the highest to the lowest observation represented in the plot. Source data are provided as a Source Data file.

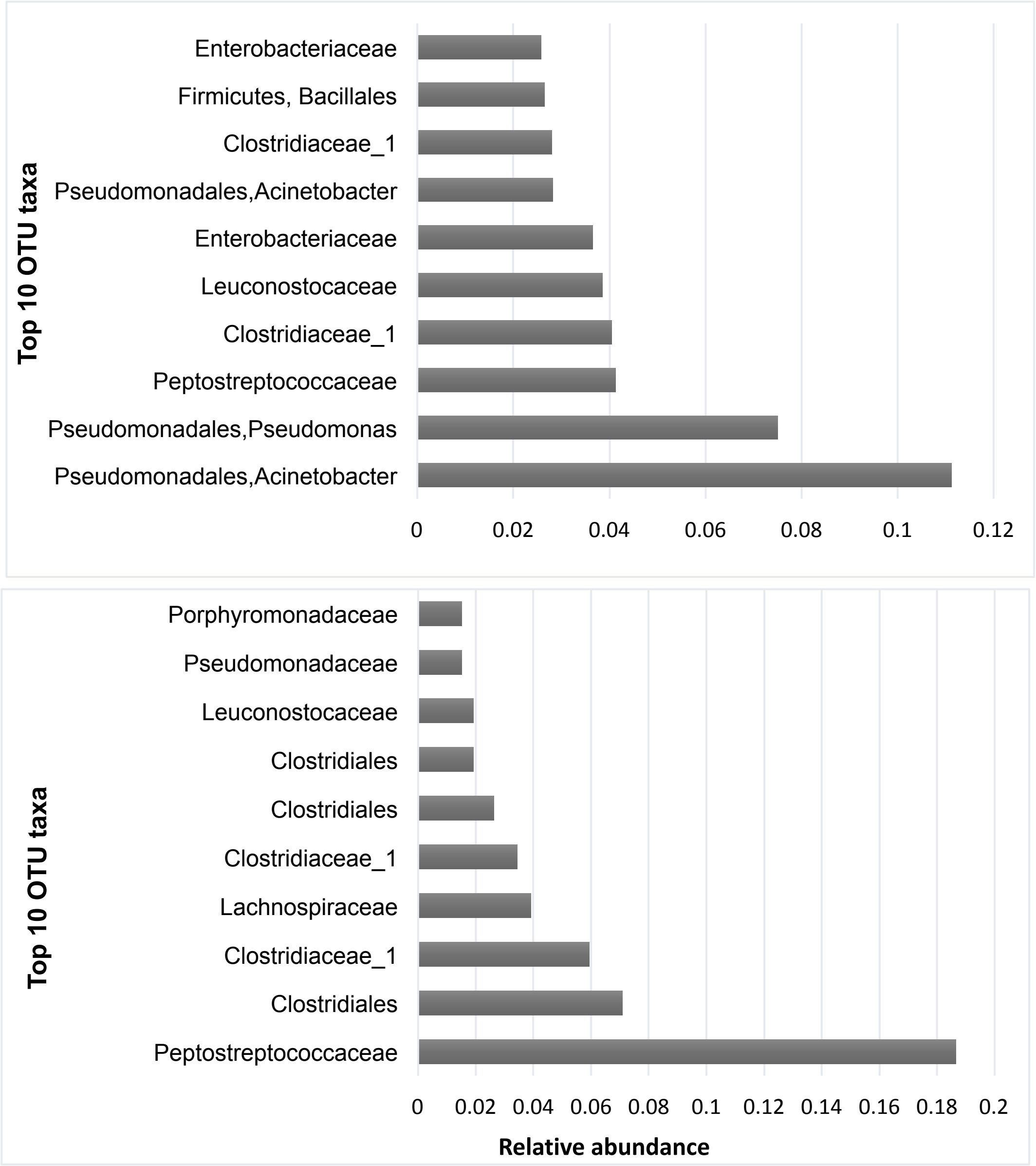

Supplementary Figure 8. Relative abundance for the top ten most prevalent bacterial families in the URT among infected and uninfected ferrets. The relative abundance was determined based on the Bayesian posterior predictive probabilities from the Infinite Dirichlet multinomial mixture models run over 1000 iterations. Analysis were performed on pyrosequencing data obtained for the V1-V3 region of the 16S rRNA of nasal wash samples obtained from 7 ferrets infected (top) with the A/Netherlands/602/2009 H1N1 virus and from

uninfected ferrets (bottom) at the time points indicated on Fig. 4. Source data are provided as a Source Data file.

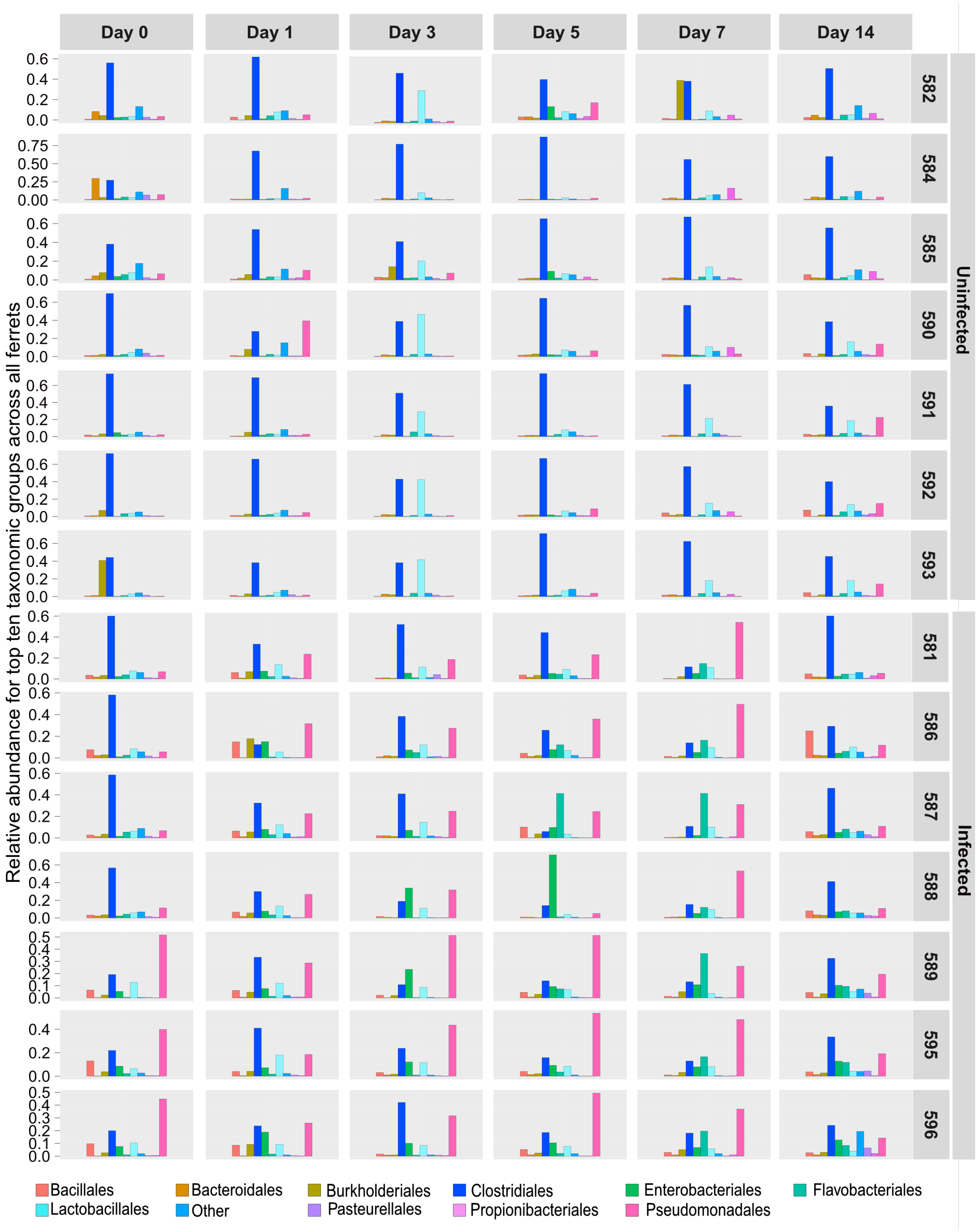
Day 0
Day 1
Day 3
Day 5
Day 7
Day 14
582
584
585
590
591
592
593
581
586
587
588
589
595
596
Uninfected
Infected
Relative abundance for top ten taxonomic groups across all ferrets
Bacillales
Bacteroidales
Burkholderiales
Clostridiales
Enterobacteriales
Flavobacteriales
Lactobacillales
Other
Pasteurellales
Propionibacteriales
Pseudomonadales

100

Supplementary Figure 9. Comprehensive taxonomic breakdown for all 14 ferrets. The plot summarizes the relative taxonomic abundances at the order level across all timepoints for taxonomic groups that are present in greater than 5% of the samples (see legend below). *Pseudomonadales* (pink) is prevalent among the infected ferrets (bottom 7), whereas Clostridiales (dark blue) is the most abundant among uninfected ferrets (top 7). Source data are provided as a Source Data file.

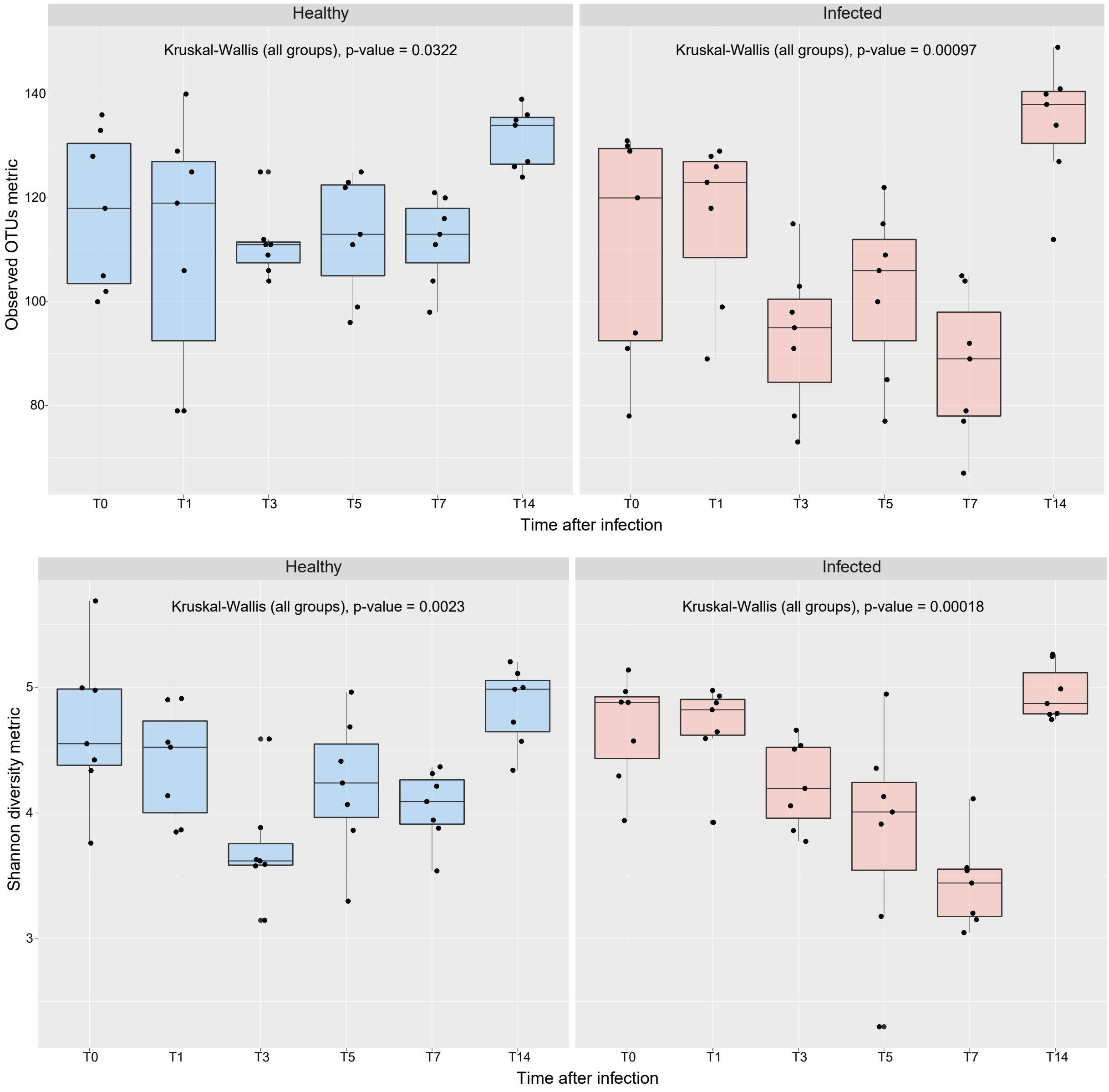

Supplementary Figure 10. Temporal diversity distance analyses of the microbiome of infected and uninfected ferrets. Changes in alpha diversity within the uninfected ferrets (n=7, blue) and infected ferrets (n=7, red) during IAV infection. A decrease in alpha diversity was observed among the infected animals during the acute phase of viral infection (3 to 7 dpi), with an eventual recovery. This was in agreement with the Pseudomonas bloom observed and the peak IAV titers collected from the same time points. The boxplots represent the diversity between the different time points. Alpha diversity was calculated using the observed OTUs

metric (A) and shannon diversity index (B). Statistical analysis and significance testing was done using the one-way Kruskal-Wallis method (significance level of 0.05). The box represents the interquartile range, the horizontal line within the box indicates the median for each sample grouping, observations are indicated by dots, and the whiskers outside the box extend from the highest to the lowest observation represented in the plot. Source data are provided as a Source Data file.

Supplementary Table 1. Clinical-epidemiological characteristics of the hospitalized human patients diagnosed with Influenza A-like illness, and healthy controls.

Characteristic	Hospitalized patients			Healthy controls (n=22)*
	Total (n=28)	H1N1 positive (n=13)	H3N2 positive (n=15)	
**Age**				
< 2 years	2	1	1	0
2 - 65 years	17	9	8	22
> 65 years	9	3	6	0
**Gender**				
Male	13	7	6	10
Female	15	6	9	12
**Clinical severity factors**				
Hospitalized by Influenza	23	8	13	N/A
CCU by Influenza	11	5	6	N/A
O2 supply	20	8	10	N/A
MV supply	7	5	1	N/A
VAD supply	5	4	1	N/A
**Vaccination and Treatments**				
Influenza Vaccine	6	2	4	5
Antibiotics	27	12	13	N/A
Antiviral	29	12	15	N/A
**Comorbidities**				
Asthma	2	0	2	N/A
COPD/Respiratory pediatric disease	3	2	1	N/A
Diabetes	8	3	4	N/A
Obesity	7	3	4	N/A
Cancer	4	3	1	N/A
Cronical cardiovascular disease	12	5	6	N/A
Cronical renal disease	2	2	0	N/A
Neurological disorder	5	2	3	N/A
Severe inmunological compromise	9	5	4	N/A
**Symptoms**				
Fever	24	12	10	N/A
Runny nose	20	9	10	N/A
Throat pain	4	1	3	N/A
Expectoration	22	11	10	N/A
Myalgia	16	8	8	N/A
Conjunctivitis	5	5	0	N/A
**Nasopharyngeal samples sequenced [a]**				
2 days	3	1	0	0
3 days	4	3	0	0
4 days	6	5	0	1
5 days	12	4	8	1
6 days	5	0	5	20
7 days	3	0	2	0
22 days	18	1	15	22

CCU: Clinical Care Unit, MV: Mechanical ventilation, VAD: Vasoactive drugs, COPD: Chronic obstructive pulmonary disease. N/A: Not applicable. [a] Days since onset of symptoms. * Healthy controls were obtained at the outpatient clinic. Source data are provided as a Source Data file.

Supplementary Table 2. Two-sided Student's two sample t test results for human samples. Comparison of every pair of boxplots (Supplementary Figure 1) to determine if they are significantly different from each other. The significance indicates that samples within the same infection state are significantly more similar to each other than samples across or between infection states. Source data are provided as a Source Data file.

Group 1	Group 2	t statistic	Parametric p-value	Parametric p-value (Bonferroni-corrected)
Flu negative vs. Flu negative	All within InfectionResult	-48.874895	0	0
Flu negative vs. Flu negative	P vs. P	-62.752559	0	0
Flu negative vs. Flu negative	P vs. U	-125.7211	0	0
Flu negative vs. Flu negative	U vs. U	-144.01251	0	0
Flu negative vs. Flu negative	Flu negative vs. P	-184.67231	0	0
Flu negative vs. Flu negative	All between InfectionResult	-239.67117	0	0
Flu negative vs. Flu negative	Flu negative vs. U	-255.04846	0	0
All within InfectionResult	P vs. P	-20.460236	1.27E-91	3.55E-90
All within InfectionResult	P vs. U	-51.51848	0	0
All within InfectionResult	U vs. U	-63.077323	0	0
All within InfectionResult	Flu negative vs. P	-82.583982	0	0
All within InfectionResult	All between InfectionResult	-144.77785	0	0
All within InfectionResult	Flu negative vs. U	-135.79499	0	0
P vs. P	P vs. U	-5.3872418	7.56E-08	2.12E-06
P vs. P	U vs. U	-10.356331	7.40E-25	2.07E-23
P vs. P	Flu negative vs. P	-19.193782	1.54E-79	4.31E-78
P vs. P	All between InfectionResult	-21.916458	3.11E-105	8.71E-104
P vs. P	Flu negative vs. U	-29.715216	3.14E-187	8.80E-186
P vs. U	U vs. U	-8.413988	4.74E-17	1.33E-15
P vs. U	Flu negative vs. P	-21.393038	7.71E-99	2.16E-97
P vs. U	All between InfectionResult	-30.462617	8.55E-200	2.39E-198
P vs. U	Flu negative vs. U	-42.0745	0	0
U vs. U	Flu negative vs. P	-12.041826	3.94E-33	1.10E-31
U vs. U	All between InfectionResult	-19.628053	4.38E-85	1.23E-83
U vs. U	Flu negative vs. U	-31.556094	1.14E-211	3.20E-210
Flu negative vs. P	All between InfectionResult	-5.4887437	4.09E-08	1.14E-06
Flu negative vs. P	Flu negative vs. U	-19.243122	1.34E-81	3.77E-80
All between InfectionResult	Flu negative vs. U	-18.618746	6.05E-77	1.69E-75

Supplementary Table 3. Non-parametric multivariate analysis using Anosim and Adonis tests. Examining the effect of clinical parameters (gender, age and antibiotic usage) on the infected human URT microbiomes. Source data are provided as a Source Data file.

Variable	Anosim test (*permutations=999*)	df (*n-1)*	Adonis test (*permutations=999*)	df (*n-1)*
Gender (n=2; M/F)	R statistic= 0.0101 p-value < 0.118	1	R^2 statistic= 0.009 p-value < 0.017	1
Antibiotic Usage (n=2; Y/N)	R statistic= 0.242 p-value < 0.001	1	R^2 statistic= 0.04231 p-value < 0.001	1
Age (n=38)	R statistic=0.402 p-value < 0.001	37	R^2 statistic= 0.427 p-value < 0.001	37
Vaccination status (n= 3; Y/N/NA)	R statistic=0.631 p-value < 0.001	2	R^2 statistic= 0.2456 p-value < 0.001	2

Supplementary Table 4: Random forest analysis results for the human microbiomes. Ranks range from the first few attributes predictive of the infection state, followed by the attributes that are most predictive of the data (maximum accuracy displayed in bold). Source data are provided as a Source Data file.

Rank (1-667)	Ranked attributes (OTUs)	OTU taxonomy[a]	Accuracy (%)
1st	**Otu000002**	*Bacteria;Proteobacteria; Gammaproteobacteria; Pseudomonadales*	64.00
2nd	Otu000002; **Otu000001**	*Bacteria;Proteobacteria; Alphaproteobacteria; Rhizobiales; Brucellaceae; Ochrobactrum*	64.00
3rd	Otu000002; Otu000001; **Otu000003**	*Bacteria; Proteobacteria; Gammaproteobacteria; Pseudomonadales; Pseudomonadaceae; Pseudomonas*	62.00
137th	Otu000002; Otu000001; Otu000003; Otu000006; Otu000055; Otu000035; Otu000005, etc (130 other OTUs)		**71.00**

[a] Taxonomy presented for most predictive OTU identified in bold

Supplementary Table 5. Two-sided Student's two sample t test results for ferrets. Comparison of every pair of boxplots (Supplementary Figure 4). The significance indicates that samples within the same infection state are significantly more similar to each other than samples across or between infection states. Source data are provided as a Source Data file.

Group 1	Group 2	t statistic	Parametric p-value	Parametric p-value (Bonferroni-corrected)
Inf_T14 vs. Inf_T14	All within Infection	-9.8555	3.79E-22	2.50E-20
Inf_T14 vs. Inf_T14	All between Infection	-11.5323	6.32E-30	4.17E-28
Inf_others vs. Inf_others	All within Infection	-4.39922	1.16E-05	0.000763
Inf_others vs. Inf_others	All between Infection	-20.6832	4.63E-88	3.05E-86
All within Infection	Inf_T14 vs. Inf_T0	-1.65626	0.097905	1
All within Infection	U vs. U	-3.93485	8.59E-05	0.00567
All within Infection	Inf_T0 vs. Inf_T0	0.572274	0.567235	1
All within Infection	Inf_T14 vs. U	-8.18941	5.37E-16	3.54E-14
All within Infection	Inf_T0 vs. Inf_others	-7.7768	1.39E-14	9.16E-13
All within Infection	U vs. Inf_T0	-11.5317	1.36E-29	8.99E-28
All within Infection	Inf_T14 vs. Inf_others	-10.7143	7.57E-26	5.00E-24
All within Infection	All between Infection	-28.681	1.34E-162	8.85E-161
All within Infection	U vs. Inf_others	-37.1721	2.51E-240	1.66E-238
U vs. U	All between Infection	-21.4854	1.69E-95	1.12E-93
Inf_T0 vs. Inf_T0	All between Infection	-4.62237	4.01E-06	0.000265
All between Infection	U vs. Inf_others	-10.5276	1.59E-25	1.05E-23

Supplementary Table 6. Random forest analysis results for the ferret microbiomes. Ranks range from the first few attributes predictive of the infection state, followed by the attributes that are most predictive of the data (maximum accuracy displayed in bold). Source data are provided as a Source Data file.

Rank (1-259)	Ranked attributes (OTUs)	OTU taxonomy[a]	Accuracy (%)
1st	**Otu000004**	*Bacteria;Proteobacteria; Gammaproteobacteria; Pseudomonadales; Moraxellaceae;Acinetobacter*	79.79
2nd	Otu000004; **Otu000028**	*Bacteria;Proteobacteria; Gammaproteobacteria; Enterobacteriales; Enterobacteriaceae;Enterobacter*	91.69
3rd	Otu000004; Otu000028; **Otu000017**	*Bacteria;Firmicutes; Bacilli;Bacillales; Family_XII;Exiguobacterium*	89.26
7th	Otu000004; Otu000028; Otu000017; Otu000001; Otu000027; Otu000170; Otu000008		**96.47**

[a] Taxonomy presented for most predictive OTU identified in bold
