# Supplementary material for: Microbiome disturbance and resilience dynamics of the upper respiratory tract during influenza A virus infection
Source: Nat Commun. 2020 May 21;11:2537. doi: 10.1038/s41467-020-16429-9 (PMC7242466; doi:10.1038/s41467-020-16429-9)
Supplement: Supplementary file 3 — Reporting Summary [file 41467_2020_16429_MOESM3_ESM.pdf]

nature research

Corresponding author(s): Rafael A. Medina
Christopher Dupont

Last updated by author(s): Apr 17, 2020

Reporting Summary

Nature Research wishes to improve the reproducibility of the work that we publish. This form provides structure for consistency and transparency in reporting. For further information on Nature Research policies, see Authors & Referees and the Editorial Policy Checklist .

Statistics

For all statistical analyses, confirm that the following items are present in the figure legend, table legend, main text, or Methods section.

n/a	Confirmed	
☐	☒	The exact sample size (*n*) for each experimental group/condition, given as a discrete number and unit of measurement
☐	☒	A statement on whether measurements were taken from distinct samples or whether the same sample was measured repeatedly
☐	☒	The statistical test(s) used AND whether they are one- or two-sided<br>*Only common tests should be described solely by name; describe more complex techniques in the Methods section.*
☐	☒	A description of all covariates tested
☐	☒	A description of any assumptions or corrections, such as tests of normality and adjustment for multiple comparisons
☐	☒	A full description of the statistical parameters including central tendency (e.g. means) or other basic estimates (e.g. regression coefficient) AND variation (e.g. standard deviation) or associated estimates of uncertainty (e.g. confidence intervals)
☐	☒	For null hypothesis testing, the test statistic (e.g. *F*, *t*, *r*) with confidence intervals, effect sizes, degrees of freedom and *P* value noted<br>*Give P values as exact values whenever suitable.*
☐	☒	For Bayesian analysis, information on the choice of priors and Markov chain Monte Carlo settings
☒	☐	For hierarchical and complex designs, identification of the appropriate level for tests and full reporting of outcomes
☒	☐	Estimates of effect sizes (e.g. Cohen's *d*, Pearson's *r*), indicating how they were calculated

Our web collection on statistics for biologists contains articles on many of the points above.

Software and code

Policy information about availability of computer code

Data collection	Sequence reads were de-multiplexed according to barcodes followed by trimming of both barcodes and adapter sequences. Following the initial processing of the sequence data, sequences were combined, dereplicated and aligned in mothur (version 1.36.1 48) using the SILVA template 49 (SSURef_NR99_123) and the sequences were organized into clusters of representative sequences based on taxonomy called Operational Taxonomic Units (OTU) using the UPARSE pipeline 50. In the ferrets, all except two libraries generated more than 3000 reads per sample. A total of 649,440 sequences were subsequently clustered into 259 OTUs with a sequence similarity threshold of 97% 48, a length threshold of 250 bp and an expected error threshold of 0.15. For human samples, the distribution of reads per sample was much more variable, with an average of approximately 10,000 reads per sample. A handful of under-represented samples (below read threshold of 50 reads) were removed prior to the downstream analyses. A total of 2,300,072 sequences were sorted into 707 OTUs, using the same thresholds as above and the same downstream filtering of the OTUs and samples was performed in a similar manner. Initial filtering of the samples ensured discarding samples containing less than 5 sequences. Libraries were normalized using metagenomeSeq's cumulative sum scaling method 51 to account for library size acting as a confounding factor for the beta diversity analysis. In addition to discarding singletons, OTUs that were observed fewer than 5 times in the count data were also filtered out to avoid the inflation of any contaminants that might skew the diversity estimates.
Data analysis	Principal Coordinates Analysis (PCoA) was done in QIIME 52 (version 1.9.1) and then visualized in Emperor 53 (version 0.9.51).<br>Taxonomic classification of the samples was done by classifying the representative sequences from the OTUs using mothur and the SILVA database.<br>The relative abundances for the taxonomic profiles for each subject was calculated in QIIME using summarize_taxa.py.<br>The visualization of the top ten most prevalent taxa for each of the organisms was done in R (version 3.2.2) using dplyr and reshape2 to manipulate the data and ggplot2 for generating the plots.<br>We employed an infinite dimensional generalization of the multinomial Dirichlet mixture model (iDMM) 22 which tries to model the original set of communities from the input data with additional posterior predictive probabilities (PPD) for statistical cut offs. The model was executed over 1000 iterations for all ferrets and 2000 iterations for all human patients.<br>The quantitative portion of the analysis was supplemented by performing random forest classification on the data to confirm the diagnostic results using Scikit-Learn (version 0.18.1) in Python (version 3.5.2) from Continuum Analytics Anaconda Suite.

Phylogenetic placement of OTUs, were run with reference sequences that were trimmed to extract the V4V5 region, aligned to the query OTU sequences using clustalo (v 1.2.1), following by analyses using RAxML (v 8.1.20) with 100 bootstrap iterations.

For manuscripts utilizing custom algorithms or software that are central to the research but not yet described in published literature, software must be made available to editors/reviewers. We strongly encourage code deposition in a community repository (e.g. GitHub). See the Nature Research guidelines for submitting code & software for further information.

Data

Policy information about availability of data

All manuscripts must include a data availability statement. This statement should provide the following information, where applicable:

- Accession codes, unique identifiers, or web links for publicly available datasets
- A list of figures that have associated raw data
- A description of any restrictions on data availability

Raw amplicon sequence reads generated during and analysed during the current study are available in the Sequence Read Archive (SRA) under accession number: SRP009696 [BioProject accession number: PRJNA76689, https://www.ncbi.nlm.nih.gov/sra/?term=PRJNA76689] for the ferrets and accession numbers: SRP092459 [BioProject accession number: PRJNA240559, https://www.ncbi.nlm.nih.gov/sra/?term=PRJNA240559] and SRP128464 [BioProject accession number: PRJNA240562, https://www.ncbi.nlm.nih.gov/sra/?term=PRJNA240562] for the infected and uninfected human subjects, respectively.

Field-specific reporting

Please select the one below that is the best fit for your research. If you are not sure, read the appropriate sections before making your selection.

☒ Life sciences ☐ Behavioural & social sciences ☐ Ecological, evolutionary & environmental sciences

For a reference copy of the document with all sections, see nature.com/documents/nr-reporting-summary-flat.pdf

Life sciences study design

All studies must disclose on these points even when the disclosure is negative.

Sample size	Samples from 28 hospitalized individuals with confirmed influenza virus infection and samples from 22 healthy individuals were used to evaluate the status and dynamic change of the upper respiratory tract (URT) microbiome during influenza virus infection. Similarly, 7 experimentally infected and 7 uninfected ferrets were also used to elucidate the effect of acute influenza virus infection on the URT microbiome. The microbiome sequences were organized into clusters of representative sequences based on taxonomy called Operational Taxonomic Units (OTU). Then the diversity and community composition was determined on the basis of presence or absence of the flu infection. Diagnostic OTU were computed to identify predictive ecostates that drived the clustering. The number of data points and data analyses were validated using a training dataset, that accurately predicted 5 samples left-out with ~85% accuracy, and which were computed to obtain a weight for each OTU's predictive capacity to classify the experimental state of each sample. In the case of the ferret experiment, the Animal Care and Use Committee allowed the use of 7 animals per group as the appropriate number of animals to achieve significant differences in infection outcome parameters (e.g. viral titers and body weights loss).
Data exclusions	A handful of under-represented samples (below read threshold of 50 reads) were removed prior to downstream analyses. Initial filtering of the samples ensured discarding samples containing less than 5 sequences. Libraries were normalized using metagenomeSeq's cumulative sum scaling method to account for library size acting as a confounding factor for the beta diversity analysis. In addition to discarding singletons, OTUs that were observed fewer than 5 times in the count data were also filtered out to avoid the inflation of any contaminants that might skew the diversity estimates.
Replication	We used 7 ferrets per group (7 infected and 7 uninfected) that were followed individually for 14 days, therefore this represent 7 independent biological replicates per study condition. In the case of humans, each individual represents a unique microbiome measurement obtained in a temporal manner. Hence this data is not reproducible. Nonetheless, the overall data was analyzed globally where predictive "ecostates" were determined (according to the predictive probability of the microbiome OTU for infected and uninfected individuals), which allowed the determination of "infected ecostates" and "healthy ecostates".
Randomization	Samples from individuals and ferrets were grouped according to their infection state. In both hosts one group corresponded to infected individuals and another group corresponded to an uninfected (Healthy) group. No other criteria was used for grouping samples or data for data analyses.
Blinding	All samples were blinded for initial sample processing and during the preparation of libraries and sequencing. Upon obtaining the sequences, clinical metadata was shared for in depth association analyses. Given that the sample collection and experimental design with ferrets included infected and uninfected conditions, no specific blinding was necessary during analyses. A final step was used to validate the identification of the predictive "ecostates" using 5 blinded samples left out.

Reporting for specific materials, systems and methods

We require information from authors about some types of materials, experimental systems and methods used in many studies. Here, indicate whether each material, system or method listed is relevant to your study. If you are not sure if a list item applies to your research, read the appropriate section before selecting a response.

Materials & experimental systems

n/a	Involved in the study
x	Antibodies
x	Eukaryotic cell lines
x	Palaeontology
	x Animals and other organisms
	x Human research participants
x	Clinical data

Methods

n/a	Involved in the study
x	ChIP-seq
x	Flow cytometry
x	MRI-based neuroimaging

Animals and other organisms

Policy information about studies involving animals; ARRIVE guidelines recommended for reporting animal research

Laboratory animals	Specific pathogen free six months old female ferrets (Mustela putorious furo) were used in this study.
Wild animals	Not Apllicable
Field-collected samples	Not Apllicable
Ethics oversight	The animal experiments described were performed under protocols approved by the Icahn School of Medicine at Mount Sinai Institutional Animal Care and Use Committee, adhering strictly to the NIH Guide for the Care and Use of Laboratory Animals.

Note that full information on the approval of the study protocol must also be provided in the manuscript.

Human research participants

Policy information about studies involving human research participants

Population characteristics	Individuals included in the study correspond to hospitalized patients with a confirmed influenza virus diagnosis and healthy controls (without influenza virus) with ages ranging from 1-97 years of age. All the individuals recruited were from Chile, and included 46% male and 54% females. Of these 96% corresponded to Hispanic whites and 4% were Hispanic-American Indian.
Recruitment	Between July and August of 2011 and June and September of 2012 (during the Southern Hemisphere autumn-winter season), a total of 30 hospitalized patients, diagnosed clinically with influenza-like illness (ILI), were recruited in Santiago, Chile.<br>Control samples from 22 healthy individuals, confirmed as negative against influenza A virus were obtained at the outpatient clinic with the same criteria in March to June of 2014.
Ethics oversight	Informed written consent was obtained under protocols 11-116 and 16-066, reviewed and approved by the Scientific Ethics Committee of the School of Medicine at Pontificia Universidad Catolica de Chile (PUC) .

Note that full information on the approval of the study protocol must also be provided in the manuscript.
